# 3-Ethyl-6-[3-(4-fluoro­phen­yl)-1*H*-pyrazol-4-yl]-1,2,4-triazolo[3,4-*b*][1,3,4]thia­diazole

**DOI:** 10.1107/S1600536810040067

**Published:** 2010-10-13

**Authors:** Hoong-Kun Fun, Ching Kheng Quah, Shridhar Malladi, Arun M. Isloor

**Affiliations:** a X-ray Crystallography Unit, School of Physics, Universiti Sains Malaysia, 11800 USM, Penang, Malaysia; b Organic Chemistry Division, Department of Chemistry, National Institute of Technology–Karnataka, Surathkal, Mangalore 575 025, India

## Abstract

In the title compound, C_14_H_11_FN_6_S, the 1,2,4-triazolo[3,4-*b*][1,3,4]thia­diazole ring system is essentially planar [maximum deviation = 0.022 (3) Å] and is inclined at dihedral angles of 15.00 (18) and 52.82 (16)° with respect to the pyrazole and phenyl rings. In the crystal, mol­ecules are linked into two-dimensional networks parallel to (100) *via* inter­molecular N—H⋯N and weak C—H⋯N hydrogen bonds. The crystal packing is further consolidated by weak π–π stacking inter­actions, with a centroid–centroid distance of 3.590 (2) Å. The crystal studied was an inversion twin with a 0.37 (13):0.63 (13) domain ratio.

## Related literature

For general background to and the biological activity of heterocycles bearing a triazole or 1,3,4-thia­diazole group, see: Farghaly (2004 ▶); Czarnocka *et al.* (1991 ▶); Unangst *et al.* (1992 ▶); Dhanya *et al.* (2009 ▶); Farghaly *et al.* (2006 ▶); Omar & Aboulwafa (1986 ▶). For the stability of the temperature controller used in the data collection, see: Cosier & Glazer (1986 ▶). For standard bond-length data, see: Allen *et al.* (1987 ▶).
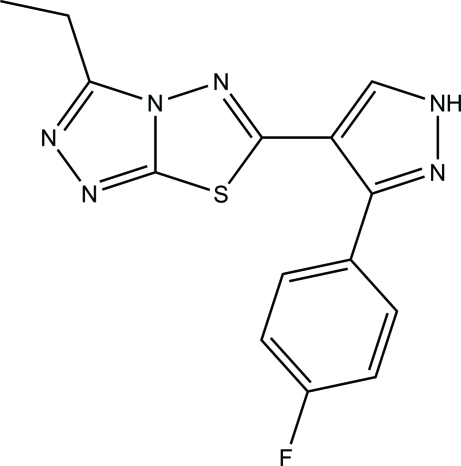


## Experimental

### 

#### Crystal data

C_14_H_11_FN_6_S
*M* *_r_* = 314.35
Orthorhombic, 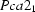

*a* = 35.053 (2) Å
*b* = 3.8463 (2) Å
*c* = 9.9482 (6) Å
*V* = 1341.26 (13) Å^3^
*Z* = 4
Mo *K*α radiation
μ = 0.26 mm^−1^
*T* = 100 K
0.64 × 0.27 × 0.09 mm

#### Data collection

Bruker SMART APEXII CCD area-detector diffractometer
Absorption correction: multi-scan (*SADABS*; Bruker, 2009 ▶) *T*_min_ = 0.852, *T*_max_ = 0.978
6132 measured reflections
2884 independent reflections
2814 reflections with *I* > 2σ(*I*)
*R*_int_ = 0.030

#### Refinement

*R*[*F* ^2^ > 2σ(*F* ^2^)] = 0.055
*wR*(*F* ^2^) = 0.123
*S* = 1.21
2884 reflections
205 parameters
1 restraint
H atoms treated by a mixture of independent and constrained refinement
Δρ_max_ = 0.45 e Å^−3^
Δρ_min_ = −0.43 e Å^−3^
Absolute structure: Flack (1983 ▶), 1283 Friedel pairs
Flack parameter: 0.37 (13)

### 

Data collection: *APEX2* (Bruker, 2009 ▶); cell refinement: *SAINT* (Bruker, 2009 ▶); data reduction: *SAINT*; program(s) used to solve structure: *SHELXTL* (Sheldrick, 2008 ▶); program(s) used to refine structure: *SHELXTL*; molecular graphics: *SHELXTL*; software used to prepare material for publication: *SHELXTL* and *PLATON* (Spek, 2009 ▶).

## Supplementary Material

Crystal structure: contains datablocks global, I. DOI: 10.1107/S1600536810040067/lh5142sup1.cif

Structure factors: contains datablocks I. DOI: 10.1107/S1600536810040067/lh5142Isup2.hkl

Additional supplementary materials:  crystallographic information; 3D view; checkCIF report

## Figures and Tables

**Table 1:** Hydrogen-bond geometry (Å, °)

*D*—H⋯*A*	*D*—H	H⋯*A*	*D*⋯*A*	*D*—H⋯*A*
N2—H1*N*2⋯N6^i^	1.03 (4)	1.95 (4)	2.899 (4)	153 (4)
C9—H9*A*⋯N5^ii^	0.93	2.50	3.368 (5)	156
C13—H13*A*⋯N1^iii^	0.97	2.49	3.419 (5)	160

Symmetry codes: (i) 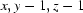; (ii) 
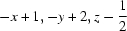
; (iii) 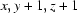.

**Fig. 1.**
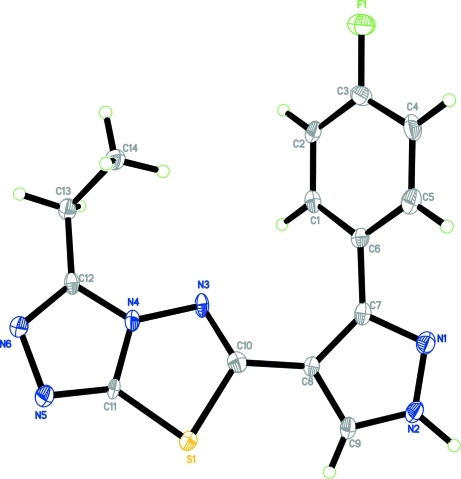
The molecular structure of the title compound showing 50% probability displacement ellipsoids for non-H atoms and the atom-numbering scheme.

**Fig. 2.**
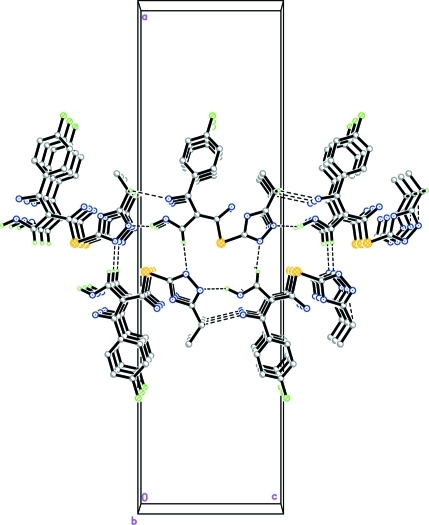
Part of the crystal structure of the title compound, viewed along the b axis. H atoms not involved in hydrogen bonds (dashed lines) have been omitted for clarity.

## supplementary crystallographic information

### Comment

The recent literature is enriched with progressive findings about the synthesis and pharmacological activity of fused heterocycles. Heterocycles bearing a triazole or 1,3,4-thiadiazole moiety are reported to show biological properties such as antibacterial (Farghaly, 2004), anti aggregatory agent (Czarnocka *et al.*, 1991), anti-inflammatory (Unangst *et al.*, 1992) and anticancer (Dhanya *et al.*, 2009) activities. In addition, the N-bridged heterocycles derived from 1,2,4-triazoles have applications in the field of medicine, agriculture and industry (Farghaly *et al.*, 2006). 1,3,4-Thiadiazoles exhibit broad spectrum of biological activities, possibly due to the presence of toxophoric N-C-S moiety (Omar & Aboulwafa, 1986). Keeping in view of the biological importance, the title compound was synthesized to study its crystal structure.

The title molecule (Fig. 1) consists of a fluorophenyl ring (F1/C1-C6), a pyrazole ring (N1/N2/C7/C8/C9) and a 3-ethyl-[1,2,4]triazolo[3,4-b] [1,3,4]thiadiazole moiety (S1/N3-N6/C10-C14). The [1,2,4]triazolo[3,4-b] [1,3,4]thiadiazole ring system is essentially planar (maximum deviation = 0.022 (3) Å for atom N4) and is inclined at angles of 15.00 (18) and 52.82 (16)° with respect to the pyrazole and phenyl rings. Bond lengths (Allen *et al.*, 1987) and angles are within normal ranges.

In the solid state, (Fig. 2), the molecules are linked into two-dimensional networks parallel to (100) *via* intermolecular N2–H1N2···N6^i^, C9–H9A···N5^ii^ and C13–H13B···N1^iii^ hydrogen bonds (see Table 1 for symmetry codes). Short intermolecular distances [3.590 (2) Å] between symmetry-related S1/N3/N4/C10/C11 (centroid Cg1) and N4-N6/C11/C12 (centroid Cg2) rings [symmetry code: X, 1+Y, Z] indicate the existence of π–π stacking interactions.

### Experimental

An equimolar mixture of 4-amino-5-ethyl-4H-1,2,4-triazole-3-thiol (0.145 g, 0.001 mol) and 3-(4-fluorophenyl)-1H-pyrazole-4-carboxylic acid (0.207 g, 0.001 mol) was dissolved in 5 ml of dry phosphorous oxychloride. The resulted solution was further heated under reflux for 7 h. Excess phosphorous oxychloride was then distilled off and the mixture was gradually poured onto crushed ice with stirring. The mixture was allowed to stand overnight and the solid was separated. The separated solid was filtered, washed thoroughly with cold water, 20% NaHCO_3_ solution and recrystallised from a mixture of dioxane and ethanol. Yield: 73.4 %. *M.p.*: 479-481 K.

### Refinement

H1N2 was located in a difference Fourier map and allowed to refined freely. The remaining H atoms were positioned geometrically and refined using a riding model with C–H = 0.93-0.97 Å and *U*_iso_(H) = 1.2 or 1.5 *U*_eq_(C). A rotating-group model was applied for the methyl group. The crystal studied was an inversion twin with a 0.37 (13) : 0.63 (13) domain ratio. The reported Flack parameter was obtained by TWIN/BASF procedure in SHELXL (Sheldrick, 2008).

### Crystal data

**Table:**

C_14_H_11_FN_6_S	*F*(000) = 648
*M**_r_* = 314.35	*D*_x_ = 1.557 Mg m^−^^3^
Orthorhombic, *P**c**a*2_1_	Mo *K*α radiation, λ = 0.71073 Å
Hall symbol: P 2c -2ac	Cell parameters from 4113 reflections
*a* = 35.053 (2) Å	θ = 2.3–30.0°
*b* = 3.8463 (2) Å	µ = 0.26 mm^−^^1^
*c* = 9.9482 (6) Å	*T* = 100 K
*V* = 1341.26 (13) Å^3^	Plate, colourless
*Z* = 4	0.64 × 0.27 × 0.09 mm

### Data collection

**Table:**

Bruker SMART APEXII CCD area-detector diffractometer	2884 independent reflections
Radiation source: fine-focus sealed tube	2814 reflections with *I* > 2σ(*I*)
graphite	*R*_int_ = 0.030
φ and ω scans	θ_max_ = 27.5°, θ_min_ = 2.3°
Absorption correction: multi-scan (*SADABS*; Bruker, 2009)	*h* = −45→38
*T*_min_ = 0.852, *T*_max_ = 0.978	*k* = −4→4
6132 measured reflections	*l* = −12→12

### Refinement

**Table:**

Refinement on *F*^2^	Secondary atom site location: difference Fourier map
Least-squares matrix: full	Hydrogen site location: inferred from neighbouring sites
*R*[*F*^2^ > 2σ(*F*^2^)] = 0.055	H atoms treated by a mixture of independent and constrained refinement
*wR*(*F*^2^) = 0.123	*w* = 1/[σ^2^(*F*_o_^2^) + (0.0155*P*)^2^ + 3.5431*P*] where *P* = (*F*_o_^2^ + 2*F*_c_^2^)/3
*S* = 1.21	(Δ/σ)_max_ < 0.001
2884 reflections	Δρ_max_ = 0.45 e Å^−^^3^
205 parameters	Δρ_min_ = −0.43 e Å^−^^3^
1 restraint	Absolute structure: Flack (1983), 1283 Friedel pairs
Primary atom site location: structure-invariant direct methods	Flack parameter: 0.37 (13)

### Special details

**Table:**

Experimental. The crystal was placed in the cold stream of an Oxford Cyrosystems Cobra open-flow nitrogen cryostat (Cosier & Glazer, 1986) operating at 100.0 (1) K.
Geometry. All esds (except the esd in the dihedral angle between two l.s. planes) are estimated using the full covariance matrix. The cell esds are taken into account individually in the estimation of esds in distances, angles and torsion angles; correlations between esds in cell parameters are only used when they are defined by crystal symmetry. An approximate (isotropic) treatment of cell esds is used for estimating esds involving l.s. planes.
Refinement. Refinement of F^2^ against ALL reflections. The weighted R-factor wR and goodness of fit S are based on F^2^, conventional R-factors R are based on F, with F set to zero for negative F^2^. The threshold expression of F^2^ > 2sigma(F^2^) is used only for calculating R-factors(gt) etc. and is not relevant to the choice of reflections for refinement. R-factors based on F^2^ are statistically about twice as large as those based on F, and R- factors based on ALL data will be even larger.

### Fractional atomic coordinates and isotropic or equivalent isotropic displacement parameters (Å^2^)

**Table:**

	*x*	*y*	*z*	*U*_iso_*/*U*_eq_	
S1	0.47232 (2)	0.8682 (2)	0.07220 (9)	0.01492 (19)	
F1	0.23063 (7)	0.4421 (7)	0.0128 (3)	0.0271 (6)	
N1	0.38932 (9)	0.3394 (9)	−0.2794 (3)	0.0178 (7)	
N2	0.42751 (9)	0.3714 (10)	−0.2988 (3)	0.0180 (7)	
N3	0.40306 (9)	0.6762 (8)	0.1424 (3)	0.0143 (7)	
N4	0.42034 (9)	0.8422 (8)	0.2497 (3)	0.0136 (6)	
N5	0.46852 (9)	1.1284 (9)	0.3395 (3)	0.0178 (7)	
N6	0.43819 (9)	1.1004 (9)	0.4319 (3)	0.0162 (7)	
C1	0.33272 (11)	0.3145 (10)	0.0202 (4)	0.0157 (7)	
H1A	0.3513	0.2268	0.0775	0.019*	
C2	0.29475 (10)	0.3088 (9)	0.0598 (4)	0.0160 (7)	
H2A	0.2876	0.2148	0.1421	0.019*	
C3	0.26802 (11)	0.4463 (11)	−0.0264 (4)	0.0185 (8)	
C4	0.27725 (12)	0.5778 (11)	−0.1507 (4)	0.0207 (9)	
H4A	0.2584	0.6626	−0.2079	0.025*	
C5	0.31518 (12)	0.5807 (12)	−0.1884 (4)	0.0204 (9)	
H5A	0.3220	0.6721	−0.2716	0.024*	
C6	0.34334 (10)	0.4494 (10)	−0.1041 (4)	0.0133 (7)	
C7	0.38318 (11)	0.4419 (10)	−0.1510 (4)	0.0136 (7)	
C8	0.41873 (10)	0.5305 (9)	−0.0900 (4)	0.0111 (7)	
C9	0.44590 (11)	0.4807 (10)	−0.1900 (4)	0.0146 (8)	
H9A	0.4720	0.5171	−0.1821	0.017*	
C10	0.42692 (10)	0.6731 (10)	0.0422 (3)	0.0147 (8)	
C11	0.45625 (11)	0.9692 (10)	0.2316 (3)	0.0118 (7)	
C12	0.40957 (10)	0.9339 (9)	0.3769 (4)	0.0123 (7)	
C13	0.37264 (10)	0.8435 (11)	0.4415 (4)	0.0153 (7)	
H13A	0.3710	0.9620	0.5273	0.018*	
H13B	0.3722	0.5954	0.4589	0.018*	
C14	0.33739 (11)	0.9399 (10)	0.3566 (4)	0.0166 (8)	
H14A	0.3147	0.8962	0.4077	0.025*	
H14B	0.3370	0.8017	0.2763	0.025*	
H14C	0.3385	1.1817	0.3331	0.025*	
H1N2	0.4397 (12)	0.306 (13)	−0.389 (4)	0.024 (13)*	

### Atomic displacement parameters (Å^2^)

**Table:**

	*U*^11^	*U*^22^	*U*^33^	*U*^12^	*U*^13^	*U*^23^
S1	0.0157 (4)	0.0175 (4)	0.0115 (4)	−0.0014 (4)	0.0014 (4)	−0.0018 (4)
F1	0.0180 (12)	0.0346 (15)	0.0287 (13)	0.0023 (11)	0.0020 (10)	0.0012 (12)
N1	0.0195 (17)	0.0204 (17)	0.0136 (16)	0.0036 (14)	−0.0003 (12)	−0.0017 (14)
N2	0.0194 (16)	0.0230 (18)	0.0117 (14)	0.0023 (15)	0.0022 (13)	−0.0031 (14)
N3	0.0209 (16)	0.0097 (16)	0.0123 (15)	0.0002 (13)	−0.0036 (13)	−0.0057 (13)
N4	0.0165 (15)	0.0151 (15)	0.0093 (13)	0.0011 (13)	−0.0026 (12)	−0.0019 (14)
N5	0.0173 (16)	0.0198 (18)	0.0163 (16)	0.0012 (14)	0.0005 (13)	−0.0056 (14)
N6	0.0165 (15)	0.0197 (18)	0.0124 (14)	−0.0003 (14)	−0.0001 (12)	−0.0019 (14)
C1	0.0189 (18)	0.0148 (18)	0.0133 (16)	0.0005 (15)	−0.0039 (14)	−0.0022 (15)
C2	0.0217 (17)	0.0153 (18)	0.0108 (17)	−0.0031 (14)	0.0016 (16)	−0.0029 (17)
C3	0.0142 (18)	0.021 (2)	0.0204 (19)	0.0006 (16)	−0.0016 (16)	−0.0025 (17)
C4	0.023 (2)	0.024 (2)	0.0146 (19)	0.0035 (18)	−0.0070 (15)	−0.0009 (17)
C5	0.025 (2)	0.021 (2)	0.0151 (18)	−0.0004 (18)	0.0005 (16)	−0.0035 (16)
C6	0.0158 (17)	0.0099 (17)	0.0142 (18)	−0.0005 (14)	0.0006 (14)	−0.0048 (14)
C7	0.0198 (18)	0.0101 (18)	0.0108 (17)	0.0018 (14)	−0.0018 (14)	0.0002 (15)
C8	0.0152 (17)	0.0080 (17)	0.0102 (16)	0.0012 (13)	−0.0007 (13)	−0.0005 (14)
C9	0.0199 (19)	0.013 (2)	0.0105 (16)	−0.0015 (15)	−0.0011 (15)	−0.0013 (14)
C10	0.0151 (17)	0.0125 (17)	0.017 (2)	0.0032 (14)	−0.0025 (13)	−0.0007 (15)
C11	0.0173 (17)	0.0112 (17)	0.0069 (16)	0.0003 (14)	−0.0004 (13)	−0.0031 (14)
C12	0.0177 (17)	0.0082 (17)	0.0109 (16)	0.0005 (14)	−0.0037 (13)	−0.0009 (14)
C13	0.0194 (18)	0.0132 (18)	0.0132 (17)	−0.0046 (15)	0.0008 (14)	0.0001 (15)
C14	0.0169 (18)	0.0162 (19)	0.0166 (18)	−0.0017 (15)	0.0017 (15)	−0.0019 (16)

### Geometric parameters (Å, °)

**Table:**

S1—C11	1.727 (3)	C2—H2A	0.9300
S1—C10	1.784 (4)	C3—C4	1.374 (6)
F1—C3	1.367 (4)	C4—C5	1.382 (6)
N1—C7	1.354 (5)	C4—H4A	0.9300
N1—N2	1.358 (4)	C5—C6	1.390 (5)
N2—C9	1.328 (5)	C5—H5A	0.9300
N2—H1N2	1.02 (5)	C6—C7	1.473 (5)
N3—C10	1.301 (5)	C7—C8	1.427 (5)
N3—N4	1.383 (4)	C8—C9	1.390 (5)
N4—C11	1.362 (5)	C8—C10	1.453 (5)
N4—C12	1.367 (5)	C9—H9A	0.9300
N5—C11	1.309 (5)	C12—C13	1.486 (5)
N5—N6	1.409 (4)	C13—C14	1.542 (5)
N6—C12	1.310 (5)	C13—H13A	0.9700
C1—C2	1.388 (5)	C13—H13B	0.9700
C1—C6	1.391 (5)	C14—H14A	0.9600
C1—H1A	0.9300	C14—H14B	0.9600
C2—C3	1.376 (5)	C14—H14C	0.9600
			
C11—S1—C10	87.55 (17)	N1—C7—C6	117.1 (3)
C7—N1—N2	105.3 (3)	C8—C7—C6	133.5 (3)
C9—N2—N1	113.0 (3)	C9—C8—C7	105.1 (3)
C9—N2—H1N2	126 (2)	C9—C8—C10	124.3 (3)
N1—N2—H1N2	121 (2)	C7—C8—C10	130.3 (3)
C10—N3—N4	108.3 (3)	N2—C9—C8	107.1 (3)
C11—N4—C12	106.6 (3)	N2—C9—H9A	126.4
C11—N4—N3	117.9 (3)	C8—C9—H9A	126.4
C12—N4—N3	135.4 (3)	N3—C10—C8	124.7 (3)
C11—N5—N6	104.5 (3)	N3—C10—S1	116.2 (3)
C12—N6—N5	110.0 (3)	C8—C10—S1	119.1 (3)
C2—C1—C6	121.0 (4)	N5—C11—N4	111.3 (3)
C2—C1—H1A	119.5	N5—C11—S1	138.7 (3)
C6—C1—H1A	119.5	N4—C11—S1	110.0 (3)
C3—C2—C1	118.0 (4)	N6—C12—N4	107.5 (3)
C3—C2—H2A	121.0	N6—C12—C13	127.0 (3)
C1—C2—H2A	121.0	N4—C12—C13	125.5 (3)
F1—C3—C4	119.1 (4)	C12—C13—C14	113.9 (3)
F1—C3—C2	118.1 (4)	C12—C13—H13A	108.8
C4—C3—C2	122.8 (4)	C14—C13—H13A	108.8
C3—C4—C5	118.3 (4)	C12—C13—H13B	108.8
C3—C4—H4A	120.9	C14—C13—H13B	108.8
C5—C4—H4A	120.9	H13A—C13—H13B	107.7
C4—C5—C6	121.1 (4)	C13—C14—H14A	109.5
C4—C5—H5A	119.5	C13—C14—H14B	109.5
C6—C5—H5A	119.5	H14A—C14—H14B	109.5
C5—C6—C1	118.8 (3)	C13—C14—H14C	109.5
C5—C6—C7	119.3 (3)	H14A—C14—H14C	109.5
C1—C6—C7	121.8 (3)	H14B—C14—H14C	109.5
N1—C7—C8	109.4 (3)		
			
C7—N1—N2—C9	1.3 (5)	C10—C8—C9—N2	−175.5 (4)
C10—N3—N4—C11	1.2 (4)	N4—N3—C10—C8	−178.0 (3)
C10—N3—N4—C12	177.3 (4)	N4—N3—C10—S1	0.7 (4)
C11—N5—N6—C12	−0.7 (4)	C9—C8—C10—N3	−169.9 (4)
C6—C1—C2—C3	−1.2 (6)	C7—C8—C10—N3	16.1 (6)
C1—C2—C3—F1	−179.7 (3)	C9—C8—C10—S1	11.4 (5)
C1—C2—C3—C4	2.1 (6)	C7—C8—C10—S1	−162.5 (3)
F1—C3—C4—C5	179.8 (4)	C11—S1—C10—N3	−1.7 (3)
C2—C3—C4—C5	−2.0 (6)	C11—S1—C10—C8	177.0 (3)
C3—C4—C5—C6	1.0 (6)	N6—N5—C11—N4	−0.1 (4)
C4—C5—C6—C1	−0.2 (6)	N6—N5—C11—S1	−179.4 (4)
C4—C5—C6—C7	177.0 (4)	C12—N4—C11—N5	0.9 (4)
C2—C1—C6—C5	0.3 (6)	N3—N4—C11—N5	178.0 (3)
C2—C1—C6—C7	−176.9 (4)	C12—N4—C11—S1	−179.6 (2)
N2—N1—C7—C8	−1.4 (4)	N3—N4—C11—S1	−2.5 (4)
N2—N1—C7—C6	177.2 (3)	C10—S1—C11—N5	−178.5 (5)
C5—C6—C7—N1	−42.4 (5)	C10—S1—C11—N4	2.2 (3)
C1—C6—C7—N1	134.7 (4)	N5—N6—C12—N4	1.3 (4)
C5—C6—C7—C8	135.7 (4)	N5—N6—C12—C13	178.5 (4)
C1—C6—C7—C8	−47.2 (6)	C11—N4—C12—N6	−1.3 (4)
N1—C7—C8—C9	1.1 (4)	N3—N4—C12—N6	−177.7 (4)
C6—C7—C8—C9	−177.2 (4)	C11—N4—C12—C13	−178.6 (3)
N1—C7—C8—C10	175.9 (4)	N3—N4—C12—C13	5.0 (6)
C6—C7—C8—C10	−2.4 (7)	N6—C12—C13—C14	131.2 (4)
N1—N2—C9—C8	−0.6 (5)	N4—C12—C13—C14	−52.0 (5)
C7—C8—C9—N2	−0.3 (4)		

### Hydrogen-bond geometry (Å, °)

**Table:**

*D*—H···*A*	*D*—H	H···*A*	*D*···*A*	*D*—H···*A*
N2—H1N2···N6^i^	1.03 (4)	1.95 (4)	2.899 (4)	153 (4)
C9—H9A···N5^ii^	0.93	2.50	3.368 (5)	156
C13—H13A···N1^iii^	0.97	2.49	3.419 (5)	160

Symmetry codes: (i) *x*, *y*−1, *z*−1; (ii) −*x*+1, −*y*+2, *z*−1/2; (iii) *x*, *y*+1, *z*+1.
